# Inhaled nitric oxide: role in the pathophysiology of cardio-cerebrovascular and respiratory diseases

**DOI:** 10.1186/s40635-022-00455-6

**Published:** 2022-06-27

**Authors:** Davide Signori, Aurora Magliocca, Kei Hayashida, Jan A. Graw, Rajeev Malhotra, Giacomo Bellani, Lorenzo Berra, Emanuele Rezoagli

**Affiliations:** 1 School of Medicine and Surgery, University of Milano-Bicocca, Monza, Italy; 2 Department of Medical Physiopathology and Transplants, University of Milan, Milan, Italy; 3 Laboratory for Critical Care Physiology, Feinstein Institutes for Medical Research, Northwell Health System, Manhasset, NY USA; 4 Department of Emergency Medicine, North Shore University Hospital, Northwell Health System, Manhasset, NY USA; 5 Department of Emergency and Critical Care Medicine, Keio University School of Medicine, Tokyo, Japan; 6 Department of Anesthesiology and Operative Intensive Care Medicine, CCM/CVK Charité, Universitätsmedizin Berlin, Corporate Member of Freie Universität Berlin, Humboldt-Universität zu Berlin, and Berlin Institute of Health, Augustenburger Platz 1, 13353 Berlin, Germany; 7 ARDS/ECMO Centrum Charité, Charité, Universitätsmedizin Berlin, Berlin, Germany; 8 Division of Cardiology, Department of Medicine, Massachusetts General Hospital, Boston, MA USA; 9 Harvard Medical School, Boston, MA USA; 10 Department of Emergency and Intensive Care, San Gerardo Hospital, Monza, Italy; 11 Department of Anesthesia, Critical Care and Pain Medicine, Massachusetts General Hospital, Boston, MA USA; 12 Respiratory Care Department, Massachusetts General Hospital, Boston, MA USA

**Keywords:** Nitric oxide, Endothelial dysfunction, Pulmonary hypertension, Cardiac arrest, Hemolysis, Shunt, Blood transfusion, Cardiopulmonary bypass, Ischemia reperfusion, Brain disorder, Toxicology

## Abstract

Nitric oxide (NO) is a key molecule in the biology of human life. NO is involved in the physiology of organ viability and in the pathophysiology of organ dysfunction, respectively. In this narrative review, we aimed at elucidating the mechanisms behind the role of NO in the respiratory and cardio-cerebrovascular systems, in the presence of a healthy or dysfunctional endothelium. NO is a key player in maintaining multiorgan viability with adequate organ blood perfusion. We report on its physiological endogenous production and effects in the circulation and within the lungs, as well as the pathophysiological implication of its disturbances related to NO depletion and excess. The review covers from preclinical information about endogenous NO produced by nitric oxide synthase (NOS) to the potential therapeutic role of exogenous NO (inhaled nitric oxide, iNO). Moreover, the importance of NO in several clinical conditions in critically ill patients such as hypoxemia, pulmonary hypertension, hemolysis, cerebrovascular events and ischemia–reperfusion syndrome is evaluated in preclinical and clinical settings. Accordingly, the mechanism behind the beneficial iNO treatment in hypoxemia and pulmonary hypertension is investigated. Furthermore, investigating the pathophysiology of brain injury, cardiopulmonary bypass, and red blood cell and artificial hemoglobin transfusion provides a focus on the potential role of NO as a protective molecule in multiorgan dysfunction. Finally, the preclinical toxicology of iNO and the antimicrobial role of NO—including its recent investigation on its role against the Sars-CoV2 infection during the COVID-19 pandemic—are described.

## Take home message

Nitric oxide (NO) is a key molecule involved in the vascular homeostasis and a key player in maintaining multiorgan viability. The therapeutic role of inhaled NO ranges from cardio-cerebrovascular to respiratory diseases to antimicrobial properties.

## Introduction

In the 1980s, nitric oxide (NO, nitrogen monoxide or nitrogen oxide) was considered just a toxic molecule, an environmental pollutant found in cigarette smoke and smog. It was known to have a role in destroying the ozone layer, and as a suspected carcinogen [1] and a precursor of acid rain. However, in the early 1990s an increasing amount of evidence showed that NO is an essential player in pathophysiology of mammals. Its activity was discovered to be fundamental in the brain, arteries, immune system, liver, pancreas, uterus, peripheral nerves, and lungs. In 1992, NO was declared the “Molecule of the Year” [2] and “initiated a new chapter in biomedical research” as Prof. Sten Lindhal stated in 1998, when the Nobel Prize in Physiology and Medicine was awarded to Robert Furchgott, Louis Ignarro and Ferid Murad for discovering NO’s role as a cardiovascular molecule [3].

### NO in the environment

NO is a colorless and odorless gas, poorly soluble in water [4]. Atmospheric NO concentration ranges between 10 and 500 parts per billion (ppb). However, its concentration is estimated to rise up to 1.7 parts per million (ppm) in highly polluted areas [5]. Further, cigarettes, combustion and lightning can significantly increase NO concentration in the surrounding environment [6]. NO is an unstable gas and undergoes oxidation to more toxic nitrogen oxides (e.g., NO_2_, N_2_O_4_).

### NO delivery systems

NO can be generated and delivered in different ways [7]: 1. pressurized cylinders are the most widely used system to store NO, delivery is regulated by sensors to control the concentrations of NO and NO_2_; 2. electric NO generators produce NO from ambient air using high-voltage electrical discharge to ionize air, which leads to the formation of NO and other byproducts filtered by a scavenging system; 3. chemical generators can produce NO by the reduction of NO_2_ by ascorbic acid; 4. NO-releasing solutions, release NO under specific chemical conditions; and 5. solid nanoparticles contains either NO or an inactive NO precursor in a stable form that releases NO in a controlled manner.

Furthermore, endovenous NO-donors (e.g., nitroglycerin, sodium nitroprusside) are commercially available drugs aimed at administering NO although not selectively (i.e., into the bloodstream)—like in the case of iNO. Despite mentioning NO-donors in this review to clarify certain mechanisms of action of NO, a comprehensive description of systemic NO-donors is out of the scope of the present review and may be consulted in other scientific reports [8, 9].

### Nitric oxide synthase (NOS)

Endogenous NO is produced by nitric oxide synthases (NOS), the enzymes that catalyze nicotinamide adenine dinucleotide phosphate (NADPH) and tetrahydrobiopterin (BH4) dependent oxidation of L-arginine to L-citrulline. NO is one of the end-products of the reaction [10, 11]. The cofactor BH4 is essential for NOS to generate NO since its absence causes NOS to shift from a dimeric to a monomeric form, thus becoming uncoupled [12].

Three NOS isoforms were discovered in humans: neuronal (nNOS or NOS I), inducible (iNOS or NOS II) and endothelial (eNOS or NOS III).

eNOS is the constitutive form in endothelial cells, thus it is the main contributor to vascular NO levels in physiological conditions. eNOS is a dimer containing two identical monomers with a reductase domain for NADPH and an oxidase domain for L-arginine.

nNOS is a constitutively expressed form of NOS that was first found in neurons [13]. It is also present in other tissues including vascular smooth muscle cells, fibroblasts, endothelial cells and cardiomyocytes. nNOS activity is regulated by calcium/calmodulin interaction and it is susceptible to feedback inhibition by NO [14]. This feature guarantees pulsatile NO production instead of generating sustained low levels, a process linked to its role in synaptic transmission [15]. Moreover, recent evidence showed that nNOS-derived NO may play an important role in vascular physiology [16].

iNOS activity is mainly regulated by gene transcription and is modestly sensitive to NO-dependent autoinhibition, moreover its action is Ca^2+^-independent compared with the other forms of NOS [17]. iNOS is widely expressed in mammalian cells, particularly in immune cells (such as dendritic cells, NK cells, mast cells and phagocytic cells including monocytes, macrophages, microglia, Kupffer cells, eosinophils, and neutrophils) [18]. NO has a complex function in immune cells since it serves as an antimicrobial agent via NO-derived peroxynitrite (ONOO^–^), a reaction product of ·NO and O_2_^–^, as well as an immunomodulator via numerous pathways of lymphocyte inhibition and apoptosis [19]. Extensively studied in various pathophysiological processes [20], iNOS expression is described also in airway epithelium [21–23] under inflammatory stimuli and in blood vessels [24], where iNOS activation can lead to excess NO concentration and severe impairment of vascular function due to reduced NO sensitivity [25].

### NO physiology

The role of NO as major mediator of vasodilatation has been well established since 1987 thanks to the work of Ignarro et al. [26] and Palmer et al. [27] The groups in two independent studies identified in NO the specific molecule previously known as endothelium-derived relaxing factor (EDRF). Moreover, a variety of nitro-vasodilators (e.g., nitroglycerin, sodium nitroprusside) is responsible for smooth muscle relaxation via cGMP synthesis, a process attributed to the release of NO [28, 29]. Endothelial cells in healthy blood vessels secrete NO tonically and enhance NO production dynamically in response to an increased shear stress, by locally controlling the organ perfusion according to changes of blood flow [30].

Although discovered as a vasodilator, NO exerts an important protective role on endothelium and guarantees vascular homeostasis [31]. Precisely, NO reduces vascular smooth muscle proliferation [32], platelet aggregation [33, 34] and leukocyte binding to endothelium [35, 36]. Furthermore, NO limits oxidative phosphorylation in mitochondria, a function that may be involved in the regulation of cell bioenergetics and apoptosis [37].

**Fig. 1:**
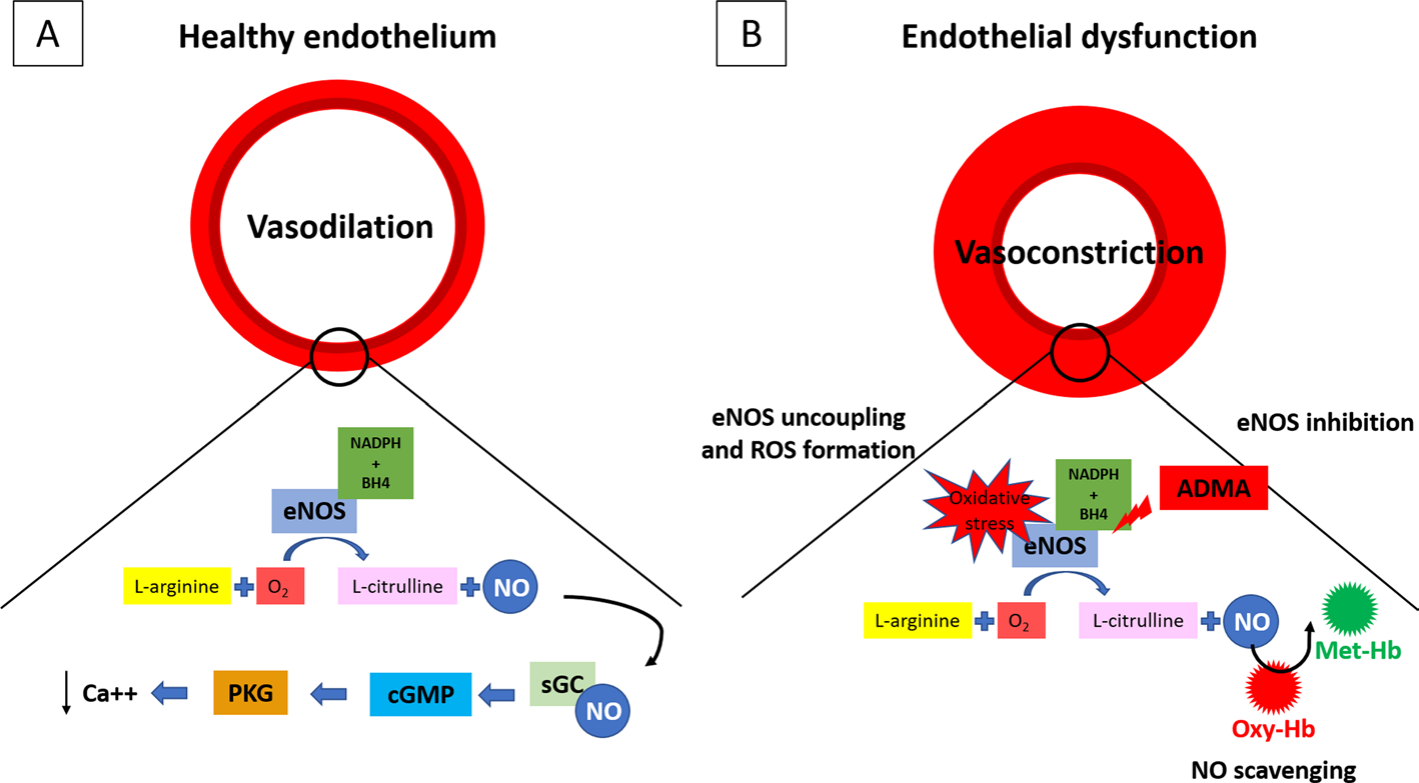
NO biosynthesis and eNOS uncoupling. Endogenous NO is produced by NOS by the oxidation of l-arginine to l-citrulline + NO (NADPH and BH4-dependent reaction). NO is one of the end-products of the reaction. Most of the effects of NO in the cardiovascular system are mediated by the activation of sGC, which catalyzes the formation of the second messenger cGMP from GTP. The activation of GMP‐dependent PKG leads to vascular relaxation (**A**). Several circumstances may alter eNOS activity causing the reduction of NO levels and triggering the production of superoxide instead of NO, a process defined as “eNOS uncoupling”. For example, the depletion of eNOS cofactor BH4, l-arginine deficiency, and increase in endogenous eNOS inhibitor ADMA lead to eNOS uncoupling. This process is largely deleterious and has been linked to endothelial dysfunction, ROS increase and other vascular pathologies. Moreover, NO bioavailability is reduced by free oxy-Hb. **B** NO: nitric oxide; NOS: nitric oxide synthase; sGC: soluble guanylate cyclase; cGMP: cyclic guanosine monophosphate; GTP: guanosine-5′-triphosphate; PKG: Protein Kinase G; BH4: tetrahydrobiopterin; ADMA: asymmetric dimethylarginine; oxy-Hb: oxyhemoglobin

Most of the effects of NO in the cardiovascular system are mediated by the activation of the enzyme-soluble guanylate cyclase (sGC), which catalyzes the formation of the second messenger cyclic guanosine monophosphate (cGMP) from guanosine-5′-triphosphate (GTP): the activation of GMP‐dependent protein kinase G (PKG) leads to vascular relaxation (Fig. 1A).

Soluble guanylate cyclase (sGC) is a heme-containing protein composed of an α and β subunits. The presence of heme results in a 100-fold increase of the enzyme activity after stimulation with NO, whereas basal enzyme activity is low without heme and does not change regardless the addition of NO [38].

NO may directly modulate other signaling systems, including nitrosylation of a wide range of proteins, thus modifying their biological activity [39]. The target proteins include the transcription factor nuclear factor kinase-B (NFκB), cell cycle-controlling proteins, and proteins involved in the generation of tissue factor [40]. Moreover, since NO is rapidly sequestered from the circulation, bound and inactivated via redox activity to nitrate (NO_3_^−^) by heme-iron of hemoglobin (Hb) [41], an additional mechanism to preserve NO bioavailability is necessary: NO is activated in vivo, requiring oxidation of NO to NO^+^, to allow its reaction with thiols [42, 43]. S-Nitrosothiols (SNO) and in particular S-nitroso-hemoglobin (SNO-Hb) are resistant to heme, thus maintaining its ability to perform vasodilatory activity [44]. Accordingly, the systemic hypoxic vasodilation observed by Guyton in the 1960s [45] is better explained by SNO-Hb itself: NO is released from SNO-Hb during deoxygenation in the microcirculation to regulate vessels directly, thus diverting blood flow to the tissues with increased oxygen demand. Furthermore, while SNO serves as the main source of NO in the microcirculation, SNO itself has a proper bioactivity that is carried out regardless sGC/PKG [46–48]. S-nitrosylation is now recognized as a fundamental post-translational modification and as a major key player in the NO bioactivity [49].

### NO toxicology

The toxicology of NO is complex since numerous NO-donors show significant side effects, particularly hypotension given their ability to vasodilate [50]. Moreover, as stated in the section on the ischemia–reperfusion syndrome (IRS), some studies showed deleterious effects of NO on brain damage [51–53].

Inhaled nitric oxide (iNO) has a complex interaction between the pharmacological properties and toxic effect [54]. Some of the toxic effects are mediated by its second messenger cGMP that, among other roles, can modulate DNA synthesis and decreases cellular proliferation. The antiproliferative effects of NO has been demonstrated in several systems, including vascular smooth muscle and human airway smooth muscle cells in vitro [55, 56]. Further studies are necessary to understand if this effect is beneficial or deleterious in hypoxic pulmonary vasoconstriction (HPV). The potential genotoxic effects of NO is also a concern since chromosomal aberrations in lung cells in rats are reported [57]. Similar results were obtained in human lymphoblastoid cells in vitro following nitric oxide treatment [58].

Nitric oxide also reacts with superoxide anion to form peroxynitrite (ONOO^−^), a highly reactive oxidant species [59]. Peroxynitrite can induce lipid peroxidation and inhibit mitochondrial respiration [60, 61]. Furthermore, it can also initiate DNA base modifications [62, 63]. Moreover, iNO can rapidly react with oxygen in the lung to form NO_2_, which is a potent pulmonary irritant that may alter the surfactant [64]. Trials on lambs and rats exposed to high doses of iNO (80 or 100 ppm, respectively) demonstrate surfactant dysfunction [65, 66].

iNO is able to exert its toxic effects outside the lung, despite the rapid inactivation by circulating Hb. In particular, iNO may cause vasodilation in extrapulmonary circulation [67], a process that may be related to the formation of S-nitroso-proteins that maintain NO biologically active [68]. Moreover, NO inhibits platelet aggregation and adherence to endothelial cells [69]. In rats, iNO (15 ppm) increased bleeding time and reduced platelet aggregation [70].

Finally, iNO can combine with hemoglobin to form met-Hb. Toxic levels of met-Hb are reached only when high dose of iNO are administered [71, 72], and a rapid clearance was demonstrated in rats and rabbits treated with iNO after the return to breathing air [73, 74].

### NO pathophysiology

iNO may have diverse clinical applications thanks to its ubiquitous role in organ function and viability. Despite extensive pre-clinical and clinical literature is available, a lot of work should be done yet to investigate iNO potential before targeting clinical trials. A summary of the highest level of evidence so far available about the iNO potential for clinical applications and highlights on research gaps before trialing in the absence of clinical trials in each specific area of research are reported in Table 1.

**Table 1:** Highest level of evidence so far available about the iNO potential for clinical applications and highlights on research gaps before trialing specific area of research

Clinical condition	Endpoint	Pre-clinical		Clinical	
		Small animals	Large animals	Lower evidence studies	Higher evidence studies
PPHN	PAP, PaO_2_/FiO_2_	–	–	–	iNO reduces PAP and improves oxygenation [198]
	Mortality	–	–	–	iNO reduces mortality [198]
ARDS	PaO_2_/FiO_2_	–	–	–	iNO is superior to control group [190–193]
	Mortality	–	–	n.s [190, 193]	n.a
Pulmonary Arterial Hypertension	PAP	–	–	–	iNO improves pulmonary hemodynamics [189]
Cardiac arrest	Brain and heart function	iNO prevents neurological and cardiac dysfunction [134]	–	n.s. [137]	n.a
	Mortality	iNO reduces mortality [135]	–	iNO reduces mortality [137]	n.a
Myocardial infarction	Infarct size after reperfusion	iNO decreases infarction size [144]	iNO decreases infarction size [145]	–	n.s [146]
Stroke	Infarct size	iNO reduces infarct size [111–113]	n.a	n.a	n.a
SAH	Brain ischemia	iNO reduces brain-edema formation and neuronal loss [125]	n.a	n.a	n.a
	Mortality	iNO reduces mortality and improves neurological outcome [125]	n.a	n.a	n.a
TBI	Secondary brain damage	iNO reduces secondary brain injury [131]	iNO reduces secondary brain injury [132, 133]	n.a	n.a
Hemolysis	Vasoconstriction	–	iNO prevents hemolysis induced vasoconstriction [149]	n.a	n.a
	AKI	–	iNO prevents hemolysis induced AKI [149]	n.a	n.a
CPB-associated hemolysis	AKI	–	–	–	iNO reduces CBP-associated AKI [166]
Transfusion associated hemolysis	Pulmonary vasoconstriction	–	iNO prevents old blood cell induced vasoconstriction [173]	iNO prevents old blood cell induced vasoconstriction (volunteers) [172]	n.a
Artificial blood hemolysis	Vasoconstriction	–	iNO prevents HBOC-induced vasoconstriction [152]	n.a	n.a
Organ transplantation	IR injury	iNO during ex vivo lung perfusion reduces lungs wet-to-dry ratio [99]	n.a	iNO improves liver function in orthotopic liver transplantation [103]	n.a

Lack of evidence is highlighted by orange cells, while the dash “–” refers to omitted literature because a study with a higher level of evidence is available for the endpoint. When the findings of human trials are conflicting with the data of preclinical studies, both studies are reported. The definition of “Lower evidence studies” refers to retrospective studies and pilot prospective randomized studies; the definition of “Higher evidence studies” refers to randomized controlled studies and meta-analysis

*AKI* acute kidney injury, *ARDS* acute respiratory distress syndrome, *CPB* cardiopulmonary bypass, *HBOC* hemoglobin-based oxygen carrier, *iNO* inhaled nitric oxide, *IR* ischemia–reperfusion, *n.a.* not available, *n.s.* not significant, *PAH* pulmonary artery hypertension, *PaO*_*2*_*/FiO*_*2*_ partial oxygen pressure-to-fraction of inspired oxygen ratio, *PAP* pulmonary arterial pressure, *PPHN* persistent pulmonary hypertension of the newborn, *SAH* subarachnoid hemorrhage, *TBI* traumatic brain injury

#### Endothelial function and vascular homeostasis

Endothelial dysfunction is a disorder characterized by an imbalance between vasodilating, antimitogenic and antithrombogenic molecules and others with vasoconstricting, prothrombotic, and proliferative properties [75, 76]. As already illustrated in the previous section, NO is one of the key substances involved in vasodilation, platelet aggregation, leukocyte adhesion activation and smooth muscle cell proliferation.

Several circumstances can alter eNOS activity causing the reduction of NO levels and triggering the production of superoxide instead of NO, a process defined as “eNOS uncoupling”. For example, the depletion of eNOS cofactor tetrahydrobiopterin (BH4), l-arginine deficiency and increase in endogenous eNOS inhibitor asymmetric dimethylarginine (ADMA), lead to eNOS uncoupling [77]. This process is largely deleterious and has been linked to endothelial dysfunction, ROS increase and other vascular pathologies [78]. Particularly, plasma ADMA levels are increased in humans with hypercholesterolemia, atherosclerosis, hypertension, chronic renal failure, chronic heart failure and insulin resistance [79]. Moreover, NO bioavailability is reduced by free oxy-Hb (see “18” section) (Fig. 1B).

Studies on eNOS knock-out mice showed that eNOS mediates basal vasodilation [80], promotes angiogenesis and helps wound healing [81]. Moreover, the vascular protective role of NO is confirmed by studies on eNOS polymorphisms associated with reduced NO production: an association of low NO synthesis was found with coronary spasm [82], hypertension [83], pre-eclampsia [84], diabetic nephropathy [85] and retinopathy [86], and vascular erectile dysfunction [87]. Thus, endothelial dysfunction contributes to the pathogenesis of cardiovascular disease and there is strong clinical evidence that loss of NO bioavailability is a crucial manifestation of endothelial dysfunction [88].

Several methods to measure endothelial dysfunction have been proposed based on the concept that healthy arteries dilate consequently to reactive hyperemia (flow-mediated vasodilatation) or after pharmacological stimuli. In disease states, this mechanism is reduced or absent. Since endothelial dysfunction is characterized by the inability to produce endogenous NO, to discriminate endothelium-independent from endothelium-dependent responses, exogenous NO-donors (e.g., sodium nitroprusside) or non-NO-donors vasodilators (e.g., acetylcholine (Ach), which is the molecule responsible of endogenous vascular NO production and consequent vasodilatation) can be applied. Consequently, a vasodilatory response after NO-donors and absence of response after acetylcholine is typical of endothelial dysfunction. Impaired endothelial-independent function is indeed associated with structural vascular alterations rather than changes in the endothelial function [89].

In 1993, Wessel et al. investigated whether cardiopulmonary bypass may induce pulmonary endothelial dysfunction and then lead to pulmonary hypertension in children with congenital heart disease undergoing surgical repair. To test their hypothesis, the authors explored the effects of iNO, as an endothelial-independent smooth muscle relaxant, and Ach, as an endothelial-dependent vasodilator. The authors demonstrated that Ach failed to reverse pulmonary hypertension. In contrast, iNO reversed increased pulmonary pressures bypassing the impaired endothelial signaling pathway of Ach. This confirmed the hypothesis that endothelial dysfunction seems to be the cause of the altered endogenous NO release. Furthermore, plasma levels of cGMP were unchanged after Ach infusion but increased more than threefold during pulmonary vasodilation with iNO. This finding was consistent with the hypothesized role of cGMP as the second messenger of effective smooth muscle relaxation in this process [90].

#### Ischemia–reperfusion syndrome

Hypoxia-induced release of NO is one of the major determinants of microvascular blood flow modifiers [91–93]. NO may exert this function by Hb S-nitrosylation at Cys93 of the β-chain [49]. The release of SNO from the deoxygenated structure of Hb is supported by data showing that wild-type mice exhibit elevated muscle blood flow after brief ischemia (reactive hyperemia), a mechanism markedly impaired in mice expressing Hb with a single point mutation in Cys93 of the β-chain, and so unable to carry SNO [94]. Also, recombinant Hb unable to carry NO was associated with increased cardiac injury and mortality in an animal model of myocardial infarction [95].

Since NO has homeostatic and protective roles on endothelium, several studies tried to assess the putative beneficial effect of NO on different organs that may be potentially prone to develop injury consequent to ischemia. Both NO-donors and iNOs were studied in IRS. However, while the mechanism of NO delivery of the former is easy to be understood, iNO is rapidly inactivated by Hb-mediated oxidation in the circulation (see also the section “18”) and may not reach the target organ. However, long-lived tissue metabolites may account for the preconditioning effects of iNO itself [96].

In animal models of myocardial IRS basal NO release was significantly decreased after myocardial ischemia and reperfusion compared to non-ischemic control [97]. Further detailed information about myocardial IRS and on the role of NO in myocardial protection are reported in the section “16” below.

Similar results were obtained on lung IRS in rats where iNO decreased inflammation and vascular permeability (i.e., as seen by a decrease in extravascular albumin accumulation) and prevented the increase in lung wet-to-dry weight ratio [98]. Furthermore, in a rodent model of ex vivo lung perfusion, iNO administration before and after lung retrieval improved lung function by reducing wet-to-dry weight ratio and pulmonary vascular resistance, guaranteeing better oxygenation, increasing lung tissue levels of cGMP, and by decreasing lung tissue tumor necrosis factor alpha (TNF-α) and iNOS [99].

Also, intestinal IRS in animal model may benefit from 80 ppm iNO since it abrogates IRS-induced perfusion reduction, the increase in leukocyte rolling, adhesion, and emigration, and the endothelial dysfunction [100]. Moreover, exogenous NO (both inhaled or via NO-donors) promotes hepatic tissue blood flow after reperfusion, decreases neutrophil accumulation and prevents the excessive production of iNOS in hepatic IRS in animals [101].

Despite significant effects in mammals, little evidence of beneficial effects of iNO in human models of IRS has been demonstrated. iNO seems to reduce pro-inflammatory cytokines after tourniquet application during knee surgery[102] and 80 ppm iNO significantly decreases hospital length of stay and accelerates the normalization of serum transaminases and coagulation times after orthotopic liver transplantation [103]. We further suggest that the time of iNO administration might play a key role in relation to the different pathogenetic stages of IRS. This may be crucial to interpret the findings on outcome in clinical studies.

### NO and the brain

#### Cerebral ischemia and stroke

Cerebral blood flow (CBF) is tightly regulated since neuronal activation requires large amounts of energy. Autoregulation and neurovascular coupling are the two main determinants of CBF, and both are affected by NO [104]. Autoregulation maintains CBF stable regardless of the changing of cerebral perfusion pressure. Inhibition of NO synthesis in eNOS knock-out mice results in the right shift of the hypotensive portion of the cerebral autoregulatory curve, thus impairing CBF at lower perfusion pressures [105]. Neurovascular coupling is the process by which the neurovascular unit (i.e., a functional structure composed by neurons, glial cells and blood vessels) modulates local CBF according to local metabolic demands [106]. nNOS inhibition in rats causes significant attenuation of the cerebral blood flow response to the somatosensory stimulation, suggesting disruption of neurovascular coupling [107].

Despite its fundamental role in brain physiology, NO activity in cerebral ischemia is extremely complex due to the interaction between the toxic effects of nitrates, the release of free radicals, and the neuroprotective effects on the vascular bed homeostasis [51–53]. iNOS can be stimulated by stress, inflammation, and infection. Under these conditions, NO can be generated in large quantities and has detrimental effects on the CNS increasing permeability of the blood–brain barrier [108].

The role of NO in stroke is controversial since a multitude of animal studies reported both neurotoxic and neuroprotective effects. Most of the neuroprotective effects of NO as reduction of infarct size in models of middle cerebral artery occlusion are associated with eNO [53, 105]. In contrast, the neurotoxicity is primarily related to nNOS and iNOS, by a mechanism related to the production of nitrates and the release of free radicals [109, 110]. However, in recent years evidences showed that overall NO has a predominant beneficial role in stroke [51], and iNO showed to be effective in reducing the cerebral infarct size in rodents models [111–113].

### Subarachnoid hemorrhage

An interesting implication of NO in the pathogenesis of delayed cerebral ischemia following subarachnoid hemorrhage (SAH) has been proposed, suggesting both eNOS and nNOS dysfunctions are among the mechanisms of the disease [114]. Driven by immunological and nonimmunological processes, red blood cells (RBCs) of the subarachnoid clot hemolyze resulting in delayed occurrence of cell-free Hb in the cerebrospinal fluid. Elevated concentrations of cell-free Hb in the cerebrospinal fluid are associated with a delayed ischemic neurological damage in patients with subarachnoid hemorrhage [115]. In addition, delayed cerebral ischemia is associated with reduction of NO levels in the cerebrospinal fluid [116]. The NO-scavenging effect of cell-free Hb might disrupt the endothelial NO signaling of cerebral arteries leading to vasoconstriction and consecutive delayed vasospasm [117, 118]. Systemic NO-donors have shown a role in delayed cerebral ischemia prevention in animal models [119, 120]. Furthermore, sequestration of cell-free Hb in large hemoglobin–haptoglobin complexes prevented the interaction of cell-free Hb with endothelial and tissue NO and restored physiological NO signaling in cerebral vasculature in an experimental setting [121].

Interestingly, recent findings showed that early cerebral ischemia after SAH may be due to constriction of pial arterioles [122–124]. In a rodent model of induced SAH, iNO significantly reduced early micro-vasospasms, while only having limited effect on large artery spasms. This resulted in less brain-edema formation, less hippocampal neuronal loss, mortality reduction, and improvement of neurological outcome [125].

#### Traumatic brain injury

Inappropriate inflammatory response is a major determinant in secondary brain damage after traumatic brain injury (TBI) [126]. The mechanism behind the hazardous increase of NO production in TBI is the upregulation of iNOS [127]. The exaggerated NO levels in the brain contribute to the TBI-associated glutamate cytotoxicity, including the pathogenesis of neuronal apoptosis and mitochondrial dysfunction [128].

However, opposite results were obtained in animal models of TBI. TBI may increase arginase activity, which competes with eNOS for L-arginine, thus limiting NO production [129]. Moreover, iNOS-deficient mice showed enhanced oxidative stress compared to the control group [130], suggesting the antithetical effect of this molecule in the brain.

Nevertheless, iNO exhibited a significant role in preserving cerebral autoregulation and secondary brain injury after TBI in murine [131] and porcine models [132, 133].

### NO and the cardiovascular system

#### Cardiac arrest

Cardiac arrest (CA) is the prototype of a global IRS of the whole body. Organs with a high metabolic demand—such as brain and heart—are particularly prone to IRS. As already illustrated in the former section of the review, NO seems to have a protective role in preclinical models of IRS. Similarly, several pharmacological interventions that increase NO bioavailability have been reported to improve outcomes in preclinical CA models [134]. Breathing 40 ppm NO for 23 h after potassium-induced CA in mice prevented neurological and cardiac dysfunction. Indeed, iNO attenuated brain edema as measured by magnetic resonance imaging at 24 h after resuscitation, while decreased apoptosis of hippocampal neurons and induction of inflammatory cytokines in the cortex. Moreover, treatment of mice with iNO markedly improved the survival rate from 31 to 85% compared to air breathing controls [135]. Finally, among the mechanisms responsible for the beneficial effect of NO in CA, sGC seems to be critically important since deletion of its 1α subunit abolished the protective effects of iNO on neurological function and survival after CA [135].

In addition, pharmacological prevention of the reduction of S-nitrosylated proteins in brain occurring after CA improves the survival rate in mice with ischemic brain injury [136]. In humans the literature is limited; however, in a pilot study on patients with intra-hospital CA, iNO was associated with significantly higher rates of survival, but no difference in favorable neurologic outcome was observed [137]. As a note of interest, preclinical evidence suggests that how cardiopulmonary resuscitation is delivered (i.e., mechanical versus manual chest compression) may decrease oxygenation after the return of spontaneous circulation because of lung edema [138–140]. The role of iNO in this setting to potentially improve oxygenation might be a field of future investigation.

#### Myocardial infarction

As already illustrated in the IRS section, NO deficiency is associated with tissue damage after reperfusion. This phenomenon is particularly relevant in a myocardial ischemia model [97].

Interestingly, the pharmacological correction of NO depletion has demonstrated better myocardial protection, which was defined as reduced ischemic area and neutrophil adherence, in a trial of myocardial IRS in dogs (i.e., NO-donor vs. placebo) [141]. Furthermore, myocardial IRS is exacerbated in the absence of eNOS [142] and—in contrast—the cardiomyocyte-specific eNOS overexpression protects myocardium [143].

Furthermore, iNO administered during myocardial IRS at 40–80 ppm reduces the infarct size and improves the left ventricular function in mice [144]. Similar results were obtained in a porcine model of myocardial infarction treated with iNO at 80 ppm 10’ before reperfusion during the subsequent 4 h. iNO improved the microvascular perfusion, reduced the infarct size, and reduced the myocardial leukocyte infiltration [145].

In humans, the inhalation of NO at 80 ppm for 4 h after reperfusion in STEMI did not reduce the infarct size at 48–72 h [146].

### NO and hemolysis

#### Hemolysis

Heme-containing proteins avidly bind to NO. Under physiological conditions, NO scavenging is slow because Hb is confined to inside red blood cells (RBCs). However, during intravascular hemolysis, free-Hb in plasma is able to rapidly bind to vascular NO, thus affecting vasomotor tone and consequently organ perfusion [147]. Particularly, this occurs by the di-oxygenation reaction of plasma oxy-Hb (Fe^2+^) with NO to form bio-inactive nitrate and met-Hb (Fe^3+^) [148].

In 2005, Minneci et al. demonstrated that intravascular hemolysis in a canine model produces dose-dependent systemic and pulmonary vasoconstriction [149]. In order to understand the mechanism behind the reduced vasoreactivity in the presence of free-Hb, the authors showed that the delivery of 80 ppm of iNO reverted the vasoconstrictive effect of plasma Hb. These observations indicate that the acute pulmonary and systemic vasoconstriction by intravascular hemolysis occurs secondarily to the accelerated di-oxygenation reaction of plasma oxy-Hb with NO to form bio-inactive nitrate and met-Hb. However, the concentration of plasma Hb itself is not the single parameter responsible to this effect because only the oxidizing biochemical form (oxy-Hb) is able to bind to NO, subsequently causing vasoconstriction and becoming vascular inactive as met-Hb.

**Fig. 2:**
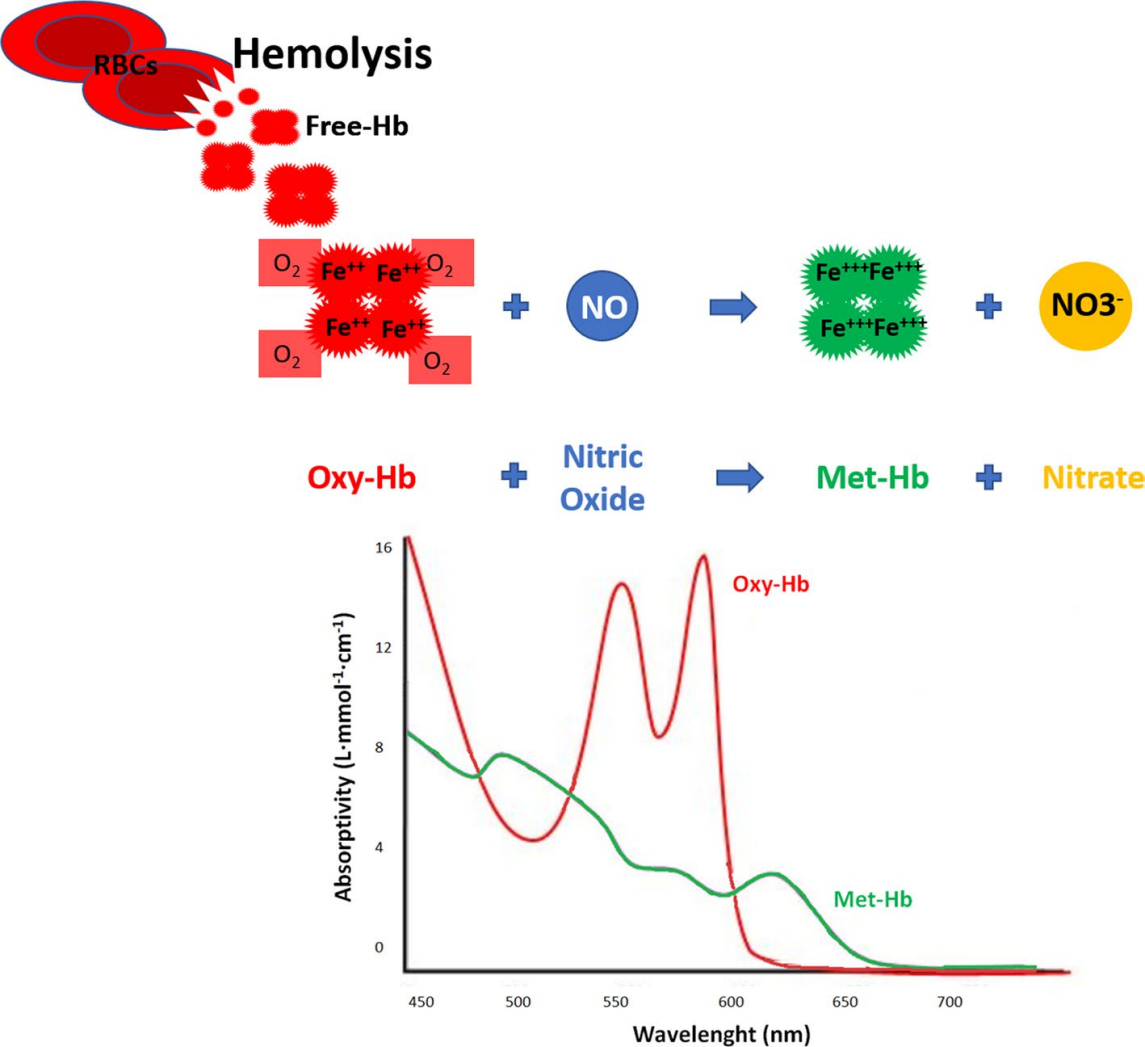
NO scavenging in hemolysis. The di-oxygenation reaction: during intravascular hemolysis in human disease, oxy-Hb (Fe^2+^) is able to rapidly bind NO, to form bio-inactive NO_3_^−^ and met-Hb (Fe3^+^). The NO scavenging causes consequently vasoconstriction. Exogenous NO can prevent this phenomenon by minimizing the scavenging of endogenous NO. The graph represents the different light absorption wavelengths of oxy-Hb and met-Hb. NO: nitric oxide; Hb: hemoglobin; RBC: red blood cell; NO3^−^: nitrate

In their same manuscript, Minneci et al. also demonstrated that the amount of hemolysis is associated with impairment of renal function assessed by a reduced creatinine clearance at 6 h from the insult. This evidence was suggested to unveil a potential link between the onset of hemolysis and organ perfusion: the greater the oxy-Hb concentration, the greater the vasoconstriction, the greater the reduction in organ perfusion with the consequent drop in creatinine clearance. Notably, creatinine clearance was restored in the hemolysis group treated with iNO, confirming its role in oxy-Hb inactivation (Fig. 2).

Intravascular sequestration of cell-free Hb by the Hb-binding protein haptoglobin was shown to protect vascular NO signaling [150]. Interestingly, in the presence of endothelial dysfunction such as in models of diabetes mellitus or hyperlipidemia in mice, haptoglobin was not able to prevent vasoconstriction [151]. Because endothelial dysfunction enhances vasoconstriction due to NO scavenging by cell-free Hb, this may suggest that in contrast to supplementation of NO, sequestration of cell-free Hb by Hb-binding molecules is not effective to prevent vasoconstriction in the presence of endothelial dysfunction [151–153].

In 2004, Gladwin et al. found an association between sickle cell disease and pulmonary hypertension, a process that may be due to NO scavenging by plasma oxy-Hb [154]; these findings were confirmed by more recent studies [155]. The protective effect of exogenous NO in sickle cell disease has been hypothesized and it may be consequent to NO restoration, red cell adhesion reduction and vaso-occlusion prevention [156]. However, the role of hemolysis in the pathogenesis of pulmonary hypertension in patients with sickle cell disease remains controversial [157].

Furthermore, pathophysiological hemodynamic changes during acute pulmonary thromboembolism may be partly caused by increased Hb decompartmentalization and consequent augmented nitric oxide consumption resulting in vasoconstriction [158].

#### Cardiopulmonary bypass-associated hemolysis

Cardio-pulmonary bypass (CPB) is known to be associated with increased pulmonary vascular resistance (PVR) [90, 159] and systemic vascular resistance (SVR) [160] particularly in the first hour after the procedure [161]. Hemolysis-induced perturbations in microcirculatory blood flow and subsequent hypoperfusion or even ischemic damage should be recognized as an important risk factor for organ injury development in patients undergoing cardiovascular surgery [162, 163].

In 2016, Rezoagli et al.[164] found that a prolonged duration of CPB (≥ 140 min) was associated with higher levels of hemolysis and both systemic and pulmonary vasoconstriction at 15 min after CPB. The investigators reported an independent linear correlation between the change of nitric oxide consumption and the change of systemic and pulmonary vascular resistance within 4 h after CPB. The length of the procedure was directly related the level of plasma Hb, and consequently to NO consumption [162], resulting in higher pulmonary and systemic vascular resistances. Reduction of NO bioavailability during CPB is not only consequent to increase in free-Hb NO consumption; also, endothelial dysfunction plays a role during this procedure impairing endogenous NO production [90].

Interestingly, the enhanced NO consumption and reduced synthesis after CPB also play a role in organ damage such as acute kidney injury (AKI) and intestinal injury [165], a process linked to the reduction of organ blood flow due to vasoconstriction already studied by Minneci as previously described. Moreover, early exogenous NO administration during CPB, improving the oxidation of oxy-Hb to met-Hb and therefore reducing systemic vasoconstriction, reduces the risk of AKI [166]. A clinical trial is currently ongoing to evaluate whether administration of 80 ppm iNO during CPB and for 24 h after surgery reduces the risk of AKI in patients with endothelial dysfunction [167].

#### Blood transfusion-associated hemolysis

Transfusion of erythrocytes stored for prolonged intervals is associated with increased morbidity (e.g., increased risk of AKI, sepsis and duration of mechanical ventilation) and mortality [168, 169]. Because storage affects the integrity of the red cell membrane, numerous erythrocytes hemolyse during storage or shortly after transfusion which is known as the so-called “storage lesion” [170, 171]. Therefore, transfusion of prolonged stored red blood cell leads to an increase of plasma Hb with consecutive scavenging of endogenous NO resulting in systemic and pulmonary vasoconstriction [151, 171, 172].

In their work, Berra et al. [172] introduced the possibility of reducing the adverse effects after transfusion of 40-day-stored packed RBCs by supplementing iNO: in obese volunteers, breathing 80 ppm NO prevented the increase of pulmonary artery pressure after transfusion of prolonged stored blood. Similarly, inhalation of 80 ppm NO prevented the vasoconstrictor response of older RBC infusions in lambs [173].

Moreover, pre-treatment of RBCs with NO-donors seems to guarantee better RBC storage quality, reducing the amount of hemolysis (measured as LDH activity) and the depletion of vital metabolites (such as 2,3-diphosphoglycerate) [174]. Similar results were obtained with RBC pre-transfusion treatment with gaseous NO or NO-donors [175].

Cell salvage devices are widely used during surgery when a consistent blood loss is expected. Hemolysis in these circumstances is a major concern due to the mechanical trauma of washing autologous blood [176, 177]. Although modern cell salvage systems can remove the majority of free-Hb during washing, they do not select between intact RBCs and damaged RBCs, which are prone to delayed hemolysis in vivo [178]. Exogenous NO may play a key role to prevent endothelial dysfunction with impaired vasorelaxation because of delayed hemolysis in vivo after administration of autologous blood by cell salvage [162].

#### Artificial hemoglobin

Blood transfusion is a common procedure performed during clinical practice and, despite all of the measures taken to ensure its safety, there are known risks associated with transfusions [178]. In addition, the use of blood products is limited by further technical issues such as product availability, need for compatibility testing, and storage and transport requirements. Moreover, there are individuals who do not accept blood transfusions. Therefore, great efforts were made to develop alternative agents that may reliably and safely replace blood. One of the most studied type of artificial blood substitute is the hemoglobin-based oxygen carrier (HBOC). HBOCs use free synthetic Hb to carry oxygen throughout the body. Due to its high toxicity, the FDA has not approved any HBOC for clinical use in the United States [179]. Since an HBOC is in fact a free-Hb complex, vasoconstriction induced by artificial blood transfusion seems to be determined by a similar scavenging mechanism of endogenous NO as in plasma-free Hb models. In 2008, Yu et al. showed in animal models that the administration of iNO could reverse systemic and pulmonary HBOC-induced vasoconstriction [152]. In 2010, the investigators further reported a relation between endothelial dysfunction and the severity of HBOC-induced side effects. Overall, these results support the hypothesis of an inverse relation between vascular NO levels and the severity of endothelial dysfunction [153].

### NO and the lungs

#### Pulmonary shunt

HPV was first identified in 1894 by Bradford [180] and later further characterized by Von Euler in 1946 [181]. HPV is the consequence of the constriction of small intrapulmonary arteries in response to alveolar hypoxia [182]: this is the cornerstone physiological mechanism owing to lung perfusion–ventilation matching. Vasoconstriction in response to hypoxia is the hallmark of the pulmonary vasculature. In contrast, systemic vessels dilate in response to hypoxia in order to increase tissue oxygen delivery [183, 184].

Extensive literature about the effect of NO during hypoxemia and HPV was provided by Professor Warren M. Zapol. The start of his scientific contributions dates back almost 50 years ago during his physiologic studies on oxygen metabolism in Weddell seals, animals that can hold their breath for over an hour on dives up to 600 m deep, tolerating a high grade of hypoxemia, high pressure and severe cold conditions [185]. The founding hypothesis was the need to treat respiratory failure to reverse hypoxia and enhance survival. However, systemic vasodilators had the opposite effect on arterial oxygenation by non-selectively dilating the pulmonary and systemic vascular bed [186]. A great advancement for the scientific community in the field of NO was the understanding of the physiological mechanism underlying its inhalation thanks to Dr. Zapol intuition. In a sheep model of thromboxane-induced and hypoxia-induced pulmonary hypertension in 1991, Dr. Frostell and Dr. Zapol with colleagues demonstrated that iNO (5–40 ppm) reversed pulmonary hypertension within 3 min; systemic vasodilation did not occur and pulmonary hypertension resumed within 3–6 min of ceasing NO inhalation [187]. Similar effects were obtained in humans with chronic pulmonary hypertension, confirming the selectivity of iNO for the pulmonary vasculature, without affecting mean systemic arterial pressure [188, 189]. iNO acts selectively on the vasculature associated with ventilated lung units: just those specific vessels that are exposed to the inhaled gas diffusing across the alveolar-capillary membrane. Selective dilatation of these vessels improves ventilation–perfusion matching. This effect has gained importance in the treatment of severe hypoxemia in patients with acute respiratory distress syndrome (ARDS) [190–193], the prototype condition of perfusion of dis-ventilated alveoli [194, 195]. Moreover, the rapid clearance of NO by Hb guarantees the absence of systemic hypotension from systemic vasorelaxation.

In 1992, Roberts et al. [196] and Kinsella et al. [197] demonstrated that iNO improved oxygenation in persistent pulmonary hypertension of the newborn (PPHN) where the lack of surfactant determines hypoxemia because of alveolar collapse and reduced ventilated alveolar units. Then, the reduction of mortality in patients with PPHN treated with iNO confirmed the robust pathophysiological link between iNO treatment and PPHN [198].

**Fig. 3:**
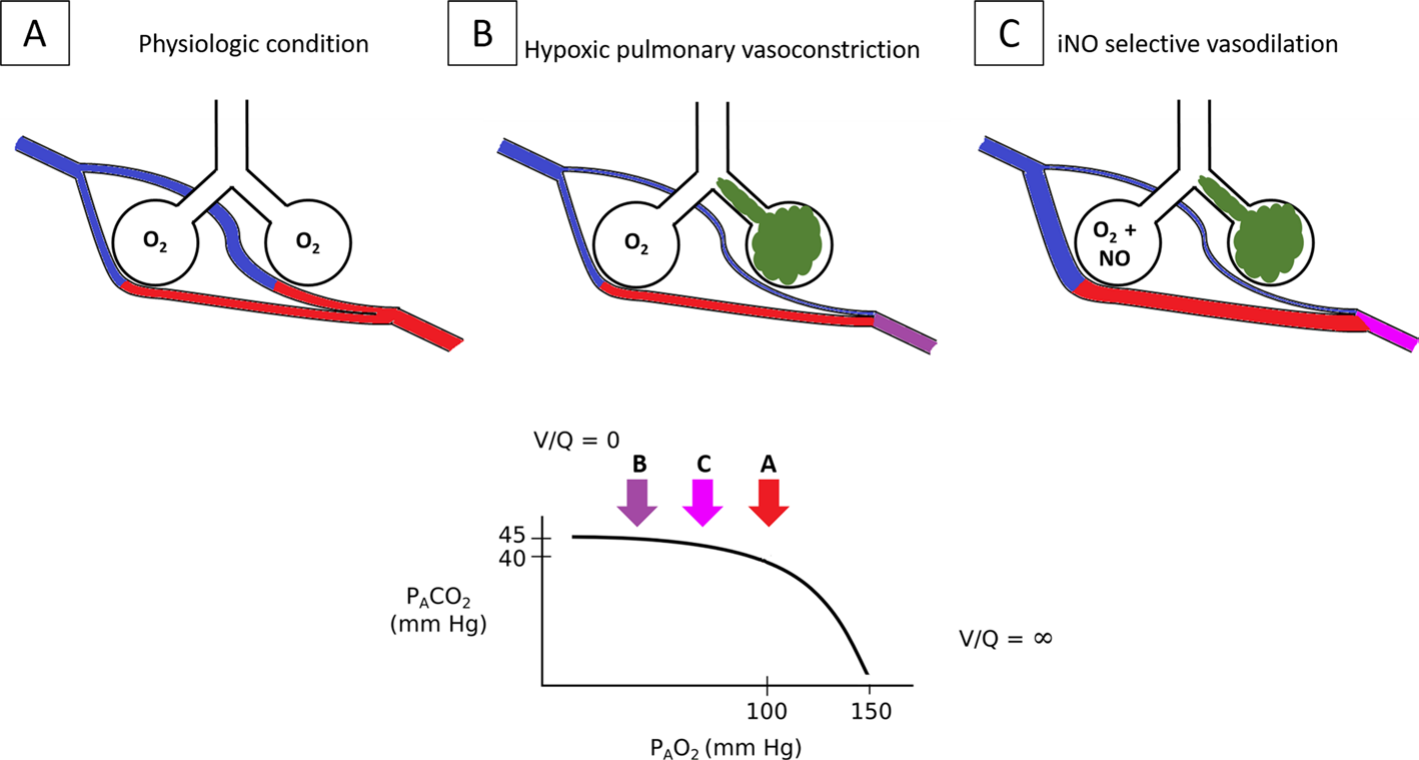
iNO reversal of hypoxemia and pulmonary hypertension during HPV. iNO is a pulmonary selective vasodilator. It diffuses selectively from ventilated alveoli to the adjacent pulmonary capillaries. This reduces PVR and the right ventricle afterload. The selective vasodilation of oxygenated vessels diverges pulmonary blood flow towards the ventilated alveoli. As a consequence, pulmonary shunt is reduced and arterial oxygenation is increased. In physiologic conditions, most of the alveoli are well ventilated and perfused, as low PVR ensures that a wide pulmonary capillary bed is recruited (**A**). If some of the alveoli are poorly or not ventilated (e.g., atelectasis, pneumonia), the pulmonary capillaries that perfuse those alveoli constrict because of HPV. The increased PVR leads to a consequent reduction of the available pulmonary vascular bed. This limits the blood perfusion of the poorly/not ventilated lung areas then limiting V/Q mismatch and pulmonary shunt (**B**). The administration of iNO in the presence of HPV increased the vasodilation of pulmonary vessels that are normally ventilated. This condition reduces PVR and reverses hypoxemia by diverging the blood flow to ventilated areas, thus reducing V/Q mismatch and pulmonary shunt (**C**). The P_A_O_2_-P_A_CO_2_ graph below, represents the partial pressure of the alveolar gases in each of the conditions previously described. In physiologic conditions, the V/Q is optimal (**A** arrow); when some the alveoli are not ventilated, hypoxemia emerges because of pulmonary shunt despite the compensatory mechanism of HPV (**B** arrow). This condition may be partially reverted by the administration of iNO (**C** arrow). The bottom panel was adapted from West JB, Luks AM. West’s Respiratory Physiology. The Essentials. Tenth Edition. Wolters Kluwer, 2015. PAO_2_: alveolar pressure of O_2_; PACO_2_: alveolar pressure of CO_2_; V/Q: ventilation–perfusion ratio; PVR: pulmonary vascular resistance; HPV: hypoxic pulmonary vasoconstriction

The In 1993, Rossaint et al. demonstrated that in patients with ARDS, a disease associated with lung heterogeneity and a high degree of right to left shunt [199], iNO reduces pulmonary vascular pressure and right ventricle overload and, by specifically dilating oxygenated vessels, iNO diverts pulmonary blood flow toward ventilated alveoli, therefore reducing pulmonary shunt and increasing arterial oxygenation [200] (Fig. 3). While hypoxemia-induced vasoconstriction reduces blood flow to non-ventilated areas therefore increasing pulmonary vascular resistance, iNO dilates vessels of better ventilated areas thus reducing pulmonary vascular resistance entering from the alveoli to the pulmonary vessels. These mechanisms are additive and allows for the reduction of pulmonary shunt and the increase of arterial oxygenation diverging pulmonary blood flow to more ventilated lung units. The selective effect of iNO for ventilated areas may be mimicked by using intratracheal NO-donors since their administration showed potential benefit on hypoxemia in ARDS in animal models, despite, no impact on mortality was reported [201, 202].

However, iNO did not show clear mortality benefits in ARDS [203]. Further, some randomized studies failed to demonstrate sustained benefits on oxygenation [191] and iNO may worsen oxygenation at high doses [204].

The use of iNO is limited in the clinical practice [205]. Current guidelines do not recommend iNO in ARDS [206] or provide a weak recommendation against its use [207], since no large phase III randomized controlled trials are available on the use of iNO in ARDS [208]. Available clinical studies in ARDS do not differentiate whether iNO may play a different role on: different etiologies [209], management [210, 211], coexisting comorbidities [212] and organ dysfunctions [213, 214] in ARDS; sex [215]; limitation of care [216]; and whether these may variably interplay on the effects of iNO on outcomes. Furthermore, not just the dose may matter [217]. The timing of iNO administration and the duration of the iNO treatment are yet to be explored [218]. This makes the clinical evidence available not conclusive so far. Hopefully, the current insights of the ARDS stratification into phenotypes—that are biologically and clinically different features within the same definition of ARDS [219, 220]—may help the understanding of the effects of numerous pharmacological treatments in ARDS including iNO [221].

### NO and sepsis

NO production is dysregulated in sepsis: exaggerated NO production may be responsible for cardiac, macrovascular, and cellular dysfunction, while reduced eNOS activity is a key factor of microvascular dysfunction. The role of NO in sepsis is not part of the current review and was extensively recently presented by Lambden [222].

### NO and COVID-19

#### SARS-CoV-2 antiviral effect of NO

NO is an antimicrobial agent. Its role was demonstrated on different viruses [223, 224] and other pathogens like bacteria, fungi, and protozoa [225–228]. NO antimicrobial activity was measured as a reduction in the cytopathic effect in vitro against SARS-CoV-1 in a concentration‐dependent manner as compared to placebo [229].

During the recent COVID-19 pandemic, the scientific productivity on SARS-CoV2 increased tremendously to better understand, treat and explore treatments that may defeat this disease [230]. Among different proposed therapeutic agents, the potential viricidal activity of NO on SARS-CoV-2 is under investigation. In vitro studies showed that the NO-donor S-nitroso-N-acetylpenicillamine inhibits SARS-CoV-2 replication. This effect correlates with both the delay and the prevention of the viral cytopathic effects in culture-type Vero E6 cells treated with NO. Akaberi and coworkers proposed that the inactivation of SARS-CoV-2 protease by S-nitrosylation is the key mechanism behind the therapeutic role of NO [231]. Other protective proposed mechanisms are the production of reactive nitrogen intermediates that inhibit the viral replication and restore the depleted endogenous NO, thus mitigating the prothrombotic and vascular complications of COVID-19 [232].

## Conclusions

NO is a molecule with a key role in human life. Its role as a beneficial agent in governing balance in organ perfusion and viability seems to overcome its limited side effects. iNOS was first demonstrated to be effective in HPV and in PPHN. However, NOS dysfunction has been linked to numerous pathologic conditions associated with the impairment of vascular homeostasis, such as ischemia, hypoxia, hemolysis, and inflammation. Furthermore, several experimental studies have shown potential beneficial effects of supplementing NO in these pathologic conditions—both as systemic NO-donors and iNO. The potential application of iNO in different organ dysfunctions is under investigation in humans. These findings may significantly improve our knowledge and understanding of the molecular pathophysiology in specific diseases, as well as in complex syndromes such as IRS and sepsis, where a specific molecular target has not been identified. The promising findings about the use of NO in preclinical research supports the translation of these results in studies aimed at exploring the effect of NO on clinical outcomes, guiding technological advances such as the optimization of organ transplantations and the use of CPB, and allowing for the safer transfusion of RBCs and HBOCs by limiting their side effects.

## Data Availability

Not applicable.

## Abbreviations

ADMA: Asymmetric dimethylarginine
AKI: Acute kidney injury
ARDS: Acute respiratory distress syndrome
CA: Cardiac arrest
CBF: Cerebral blood flow
cGMP: Cyclic guanosine monophosphate
CPB: Cardiopulmonary bypass
EDRF: Endothelium-derived relaxing factor
eNOS: Endothelial nitric oxide synthase
GTP: Guanosine-5′-triphosphate
Hb: Hemoglobin
HBOC: Hemoglobin-based oxygen carrier
HPV: Hypoxic pulmonary vasoconstriction
iNO: Inhaled nitric oxide
iNOS: Inducible nitric oxide synthase
IRS: Ischemia–reperfusion syndrome
NFkB: Nuclear factor kinase-B
nNOS: Neuronal nitric oxide synthase
NO: Nitric oxide
NOS: Nitric oxide synthase
ONOO^−^: Peroxynitrite
PAH: Pulmonary artery hypertension
PaO_2_/FiO_2_: Partial oxygen pressure-to-fraction of inspired oxygen ratio
PAP: Pulmonary arterial pressure
PKG: Protein kinase G
ppb: Parts per billion
ppm: Parts per million
PPHN: Persistent pulmonary hypertension of the newborn
PVR: Pulmonary vascular resistance
RBCs: Red blood cells
SAH: Subarachnoid hemorrhage
sGC: Soluble guanylate cyclase
SNO: S-Nitrosothiols
SNO-Hb: S-Nitroso-hemoglobin
SVR: Systemic vascular resistance
TBI: Traumatic brain injury
